# Anterior Cutaneous Nerve Entrapment Syndrome After Fotona Laser Skin Tightening Treatment: A Case Report

**DOI:** 10.1177/22925503251392570

**Published:** 2025-11-20

**Authors:** Merry Faye Graff, Alexander Platt, Brett Ponich, Aaron Knox

**Affiliations:** 1 Cumming School of Medicine, 70401 University of Calgary, Calgary, Alberta, Canada; 2 Division of Plastic and Reconstructive Surgery, 2129 University of Calgary, Calgary, Alberta, Canada; 3 Department of Surgery, 70401 University of Calgary, Calgary, Alberta, Canada

**Keywords:** anterior cutaneous nerve entrapment syndrome, abdominal pain, laser therapy, abdominoplasty, neurectomy, syndrome de compression du nerf cutané antérieur, abdominoplastie, neurectomie, traitement au laser, douleur abdominale

## Abstract

Anterior cutaneous nerve entrapment syndrome (ACNES) is an underdiagnosed cause of chronic abdominal wall pain. It results from the entrapment of the cutaneous branches of the thoracoabdominal nerves. We describe the case of a 54-year-old female who developed ACNES following Fotona laser tightening treatment of the abdomen. She underwent a neurectomy of the T6 and T10 intercostal nerves during body contouring surgery and reported complete resolution of ACNES symptoms.

## Résumé

Le syndrome de compression du nerf cutané antérieur est une cause sous-diagnostiquée de douleurs chroniques de la paroi abdominale. Il est causé par la compression des branches cutanées des nerfs thoraco-abdominaux. Les chercheurs décrivent le cas d’une femme de 54 ans qui a développé un tel syndrome après un traitement de resserrement de l’abdomen au laser Fotona. Elle a subi une neurectomie des nerfs intercostaux T6 et T10 pendant une opération de remodelage du corps et a rendu compte d’une résolution complète de ces symptômes.

## Introduction

Noninvasive body contouring and skin-tightening treatments continue to grow in popularity. Fotona TightSculpting^®^ combines the 1064 nm Nd:YAG and 2940 nm Er:YAG laser in a single system to induce hyperthermic lipolysis and stimulate collagen remodeling.^1,2^ Documented complications from this treatment include transient erythema, warmth, and tenderness, with literature indicating a generally safe profile for this procedure.^3–5^

**Figure 1.**
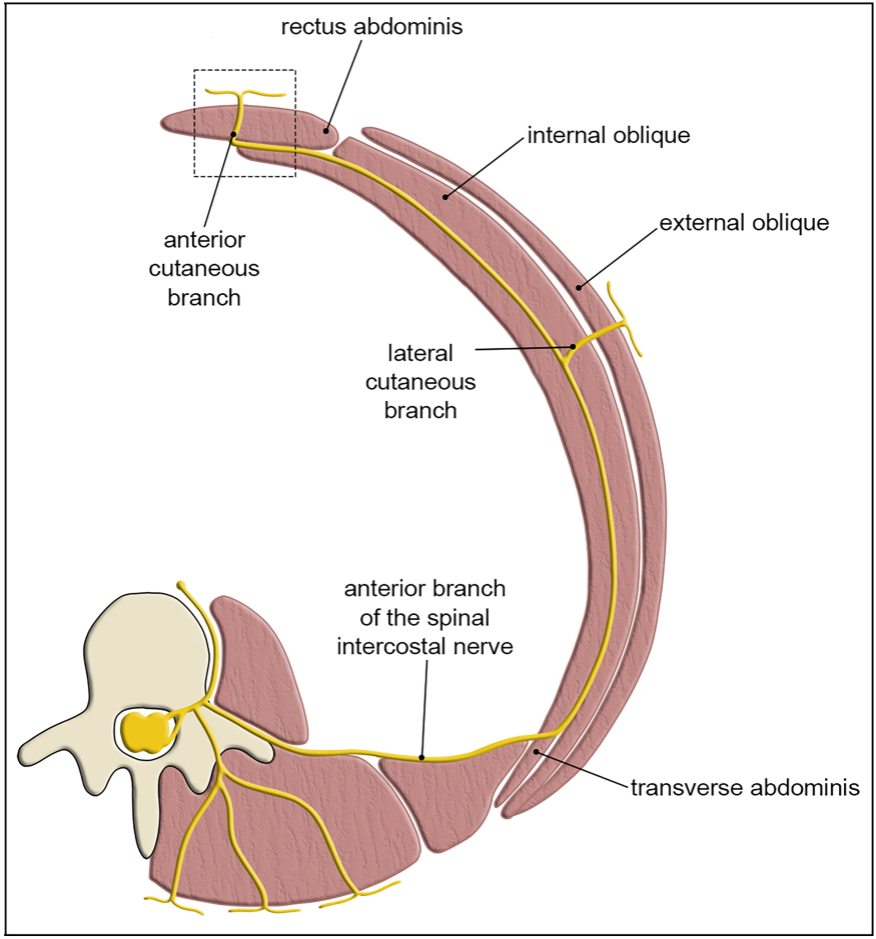
Cross-section through the thorax shows the path of the spinal nerve as it branches into the anterior cutaneous branch and traverses the rectus abdominis (box = region of entrapment). Adapted from: Smelt H, Pouwels S, Apers J, et al. Anterior Cutaneous Nerve Entrapment Syndrome: Two Case Reports of the Forgotten Diagnosis After Bariatric Surgery. Cureus. 2020;12(6):e8499. CC BY 4.0

Anterior cutaneous nerve entrapment syndrome (ACNES) is underdiagnosed, accounting for 10% to 30% of chronic abdominal wall pain in adults.^6^ It arises from the compression of one or more anterior cutaneous branches of the intercostal nerves (T7-T12), as they traverse the rectus muscle to reach the abdominal wall (Figure 1).^7^ ACNES presents with unilateral, sharp, or burning pain localized to the abdominal wall, exacerbated by movements engaging the abdominal muscles.^7^ A triad of localized pain, a positive Carnett test, and diagnostic tests that rule out other conditions supports this diagnosis.^8,9^ Conservative therapies have varying efficacy with refractory symptoms requiring anterior neurectomy to be curative.^9–13^ We present a case of ACNES following Fotona TightSculpting^®^ of the abdomen, a previously unreported etiology.

## Case Report

A 54-year-old female presented with a history of persistent right-sided abdominal wall pain that began after undergoing two 15-min sessions of abdominal Fotona TightSculpting^®^ at a professional beauty salon operating in Calgary, Alberta. She described sharp, burning pain radiating from the abdomen to the anterior ribs and flank, worsened by pressure and physical activity. This pain began immediately following her second laser treatment and worsened over several weeks. The patient reported no changes in skin laxity after either treatment, but did develop a minor superficial burn near her umbilicus following the second treatment. Her past medical history was significant for a distant open appendectomy, 2 vaginal deliveries and breast augmentation. She was otherwise healthy, a nonsmoker, and had no other history of nonsurgical body contouring procedures.

### Initial Investigations

**Figure 2.**
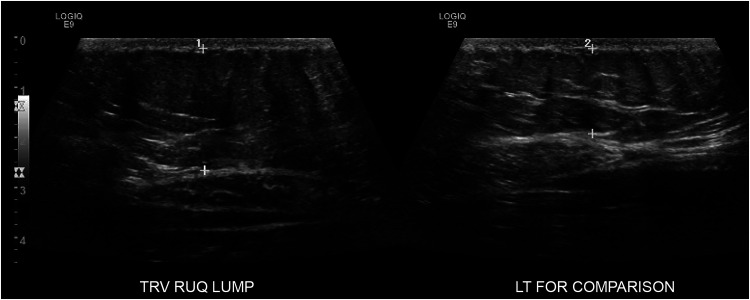
Ultrasound demonstrating region of abundant lipomatous tissue in the right upper quadrant with the left for comparison. TRV, transverse view; RUQ, right upper quadrant; LT, left.

Magnetic resonance imaging of the thoracic and lumbar spine, computed tomography of the abdomen and pelvis, and chest X-ray were all unremarkable. Abdominal ultrasound (US) was initially reported as normal. However, a second US of the abdominal wall later revealed a superficial, asymmetric region of compressible lipomatous tissue in the right upper quadrant corresponding to the area of concern (Figure 2). There were no other abnormal findings. Initially, nerve and transverse abdominis plane blocks relieved pain temporarily. During one of these treatments, the performing anesthetist observed a hyperechoic region within the rectus muscle, potentially representing an entrapment point. Based on her favorable response to local anesthetic intervention, the patient was referred to plastic surgery for possible ACNES.

### Plastic Surgery Consultation

On physical examination, the patient exhibited diffuse tenderness on the right side of the abdomen below her inframammary fold and lateral to her umbilicus. She was diagnosed with ACNES following a positive Carnett test at the right rectus abdominus along the T6 and T10 distributions. Additionally, she expressed interest in abdominoplasty. After the risks and benefits of the joint procedure were discussed, she consented and booked for abdominoplasty and neurectomy of the T6 and T10 intercostal nerves.

### Surgical Technique

**Figure 3.**
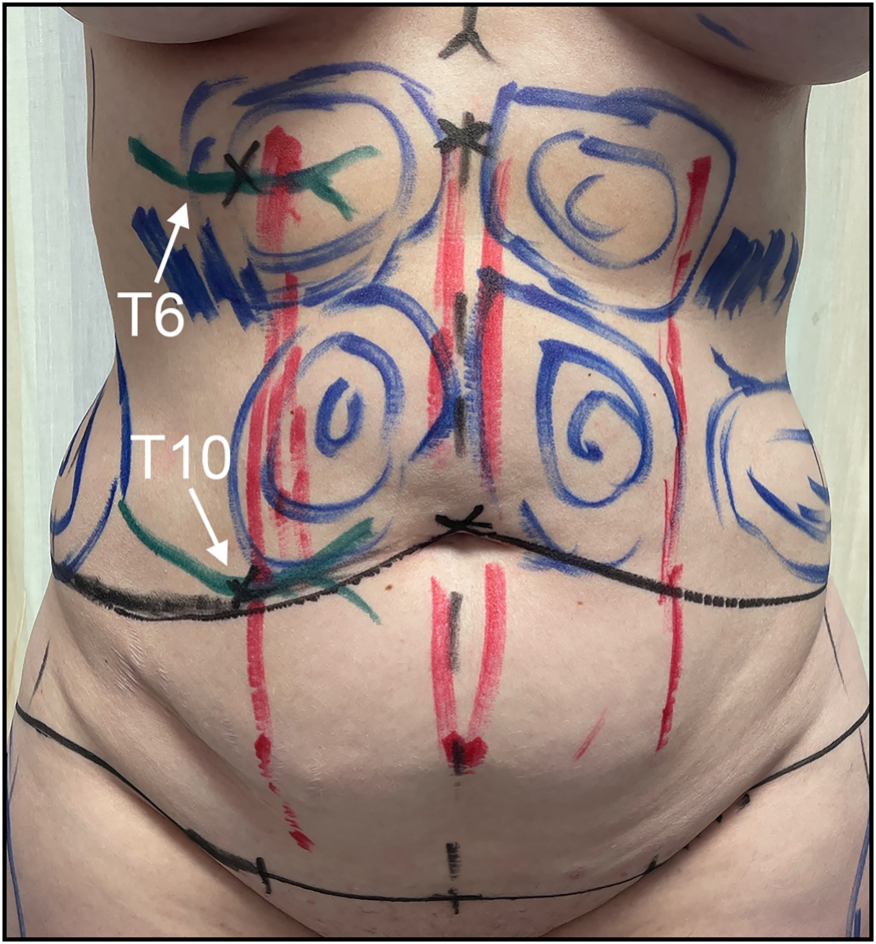
Preoperative T6 and T10 markings and the point where the anterior cutaneous branches enter the rectus abdominis (black X over green marker).

**Figure 4.**
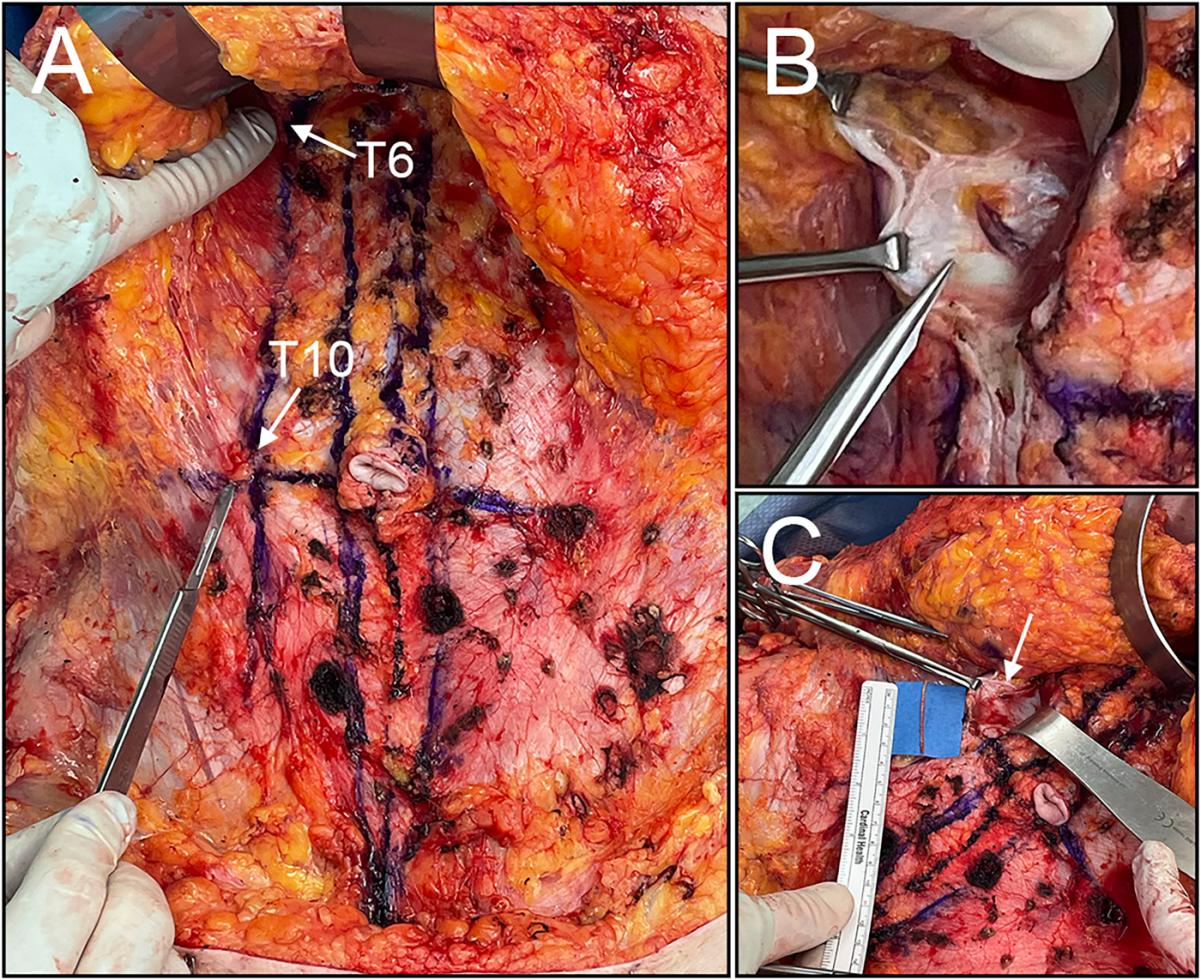
(A) Initial incision made through the skin and subcutaneous fat and raised to expose the anterior rectus sheath fascia. (B) Anterior cutaneous nerve as it passes through the posterior rectus sheath. (C) Resected nerve (arrow: excision location).

**Figure 5.**
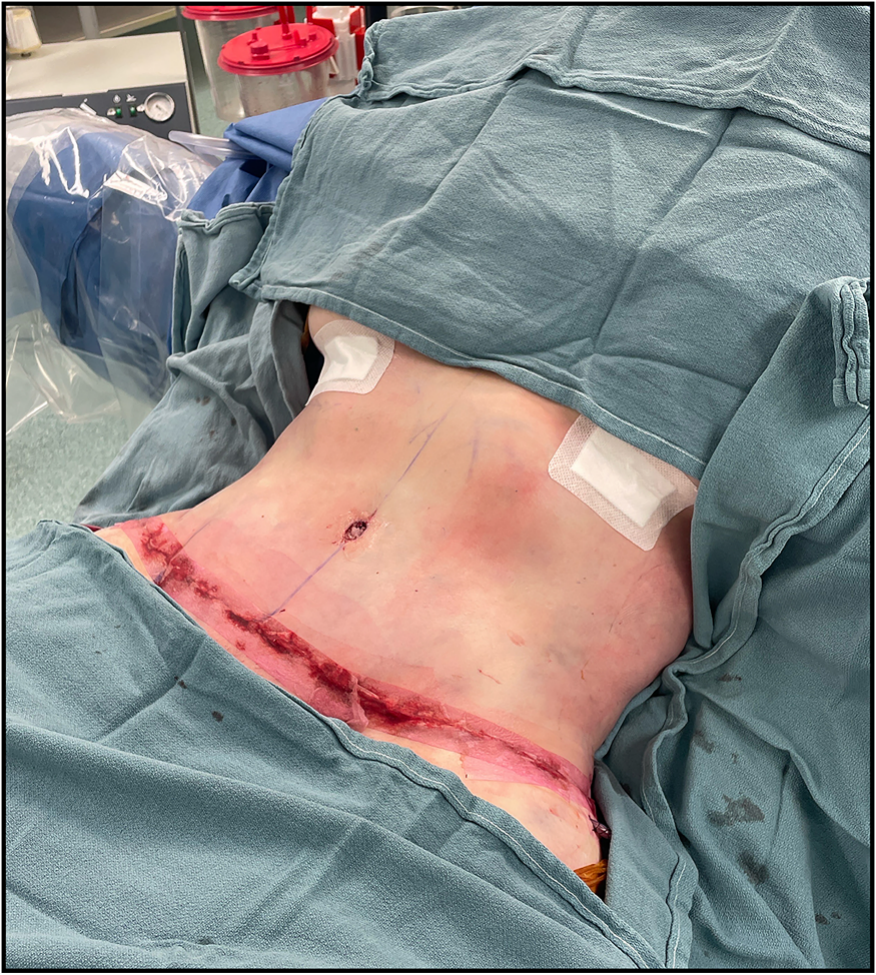
Intraoperative closure of the anterior aspect of the abdomen following the abdominoplasty.

The 2 areas of maximal pain were located and marked preoperatively (Figure 3). A transverse suprapubic incision extending to the supragluteal cleft was designed and incised. The abdominal subcutaneous tissue was raised off the rectus sheath to the level of the costal margin. The areas corresponding to her T6 and T10 intercostal nerves were then marked on the fascia (Figure 4A). The anterior rectus fascia was incised longitudinally to expose the rectus abdominis muscle. The muscle was retracted medially to visualize the intercostal nerves. T10 was identified and isolated from its associated neurovascular bundles (Figure 4B). The nerve was extracted and excised proximally, leaving the proximal nerve stump to retract (Figure 4C). This was repeated for the T6 intercostal nerve. Following the neurectomies, fascial rents were oversewn, and the abdominoplasty was carried out in standard fashion with rectus diastasis repair (Figure 5). Of note, no irregularities were noted to correspond with the lipomatous tissue previously identified on US.

**Figure 6.**
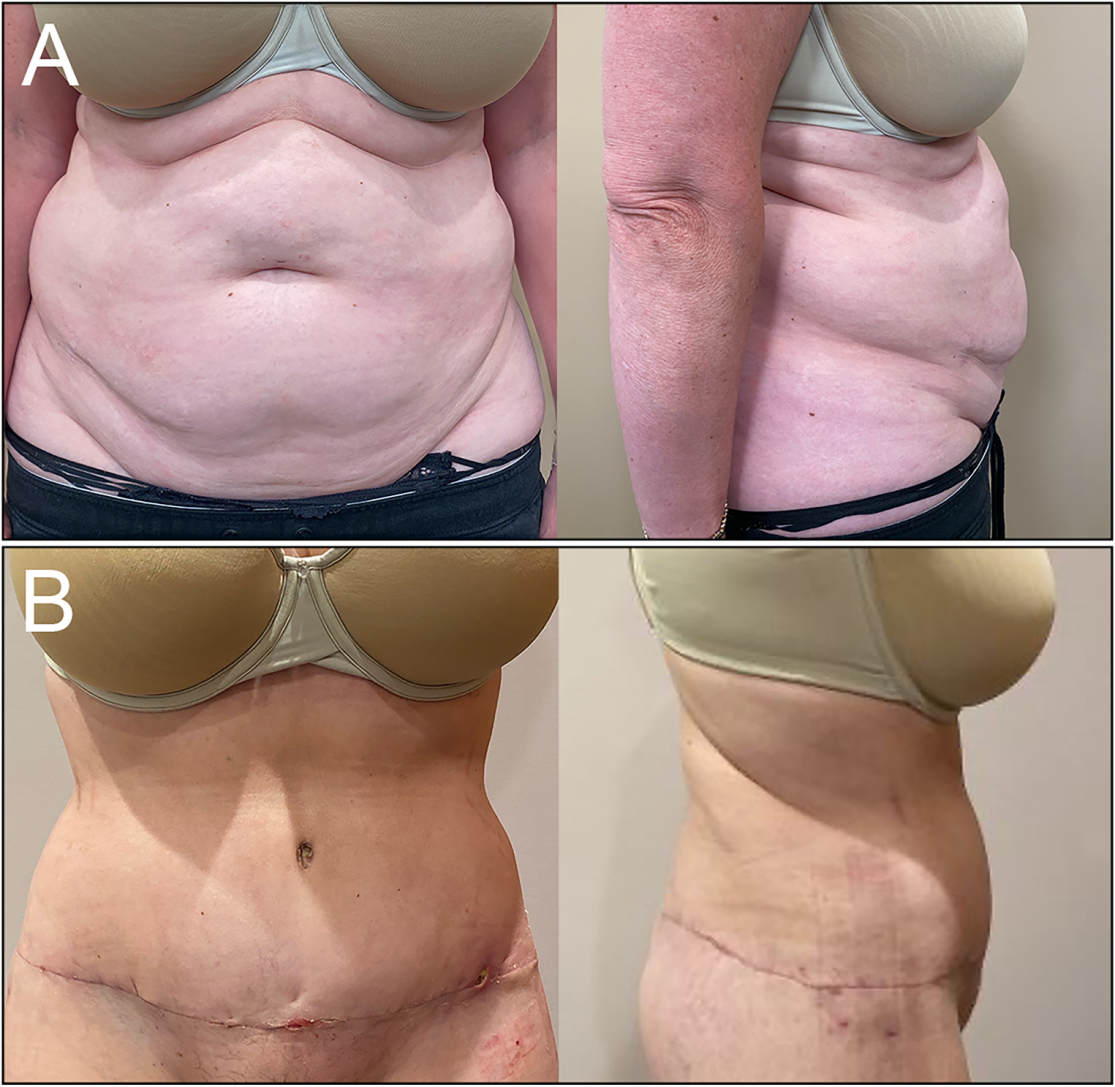
(A) Preoperative and (B) 4-week follow-up anterior and lateral views.

The patient tolerated surgery well and was discharged home from day surgery with no postoperative complications. At 4-week follow-up, the patient continued to heal well with complete resolution of her ACNES, which remained resolved 8 months postop (Figure 6).

## Discussion

The anterior cutaneous nerves are vulnerable to entrapment as they pass through a fibrous ring within the posterior sheath of the rectus abdominus muscle.^7^ Consequently, factors that alter the surrounding tissue or raise intra-abdominal pressure can cause compression and subsequent pain.^14–17^ Additionally, previous abdominal surgery increases the risk of fibrosis, scarring, and edema, which may compress these nerves.^12,18^

We describe a 54-year-old female with ACNES following Fotana laser tightening. To our knowledge, there are no other similar case reports. While this patient has a history of prior abdominal surgery and pregnancies, it is unlikely they have contributed to her nerve entrapment, given the timeline of her symptoms following laser treatment. It is important to note that ACNES is more prevalent in females and occurs more often between the ages of 20 to 30 and 40 to 50.^8,11–13^ The diagnostic process outlined in this case report highlights the importance of considering ACNES in the differential diagnosis of chronic abdominal wall pain, particularly when other findings are unremarkable.

The significance of the lipomatous tissue detected on US and its relation to Fotona treatment is uncertain. Paroxysmal adipose hyperplasia has been reported as a rare complication of other body contouring modalities, such as cryolipolysis and radiofrequency treatments.^19,20^ While TightSculpting^®^ and radiofrequency employ thermal energy to reduce fat, their mechanisms differ. However, both modalities can impact deeper tissue structures, raising the risks of unintended effects.

Although we cannot establish direct causation between TightSculpting^®^ and ACNES, it is important to recognize the potential for unintended side effects with emerging aesthetic modalities. In addition to neo-collagenesis, heat generated during laser treatment can cause tightening of deep structures such as fascia, reticular dermis, and retinaculum.^1,2^ It is possible that the thermal effects of laser treatment used in these procedures alter surrounding tissue enough to predispose patients to entrapment of the cutaneous nerves of the abdomen.

## Conclusion

We report a case of ACNES following Fotona TightSculpting^®^ of the abdomen. While this has not been a previously reported outcome of laser treatment, it is possible that the thermal effect from laser systems may increase the risk of ACNES. Early recognition of ACNES and appropriate management are essential for ensuring favorable outcomes and alleviating patient discomfort in this context.

## Supplemental Material

**Figure:** Video 1.
